# Two New Oxysporone Derivatives from the Fermentation Broth of the Endophytic Plant Fungus *Pestalotiopsis karstenii* Isolated from Stems of *Camellia sasanqua*

**DOI:** 10.3390/molecules17078554

**Published:** 2012-07-17

**Authors:** Du Qiang Luo, Lei Zhang, Bao Zhong Shi, Xiao Mei Song

**Affiliations:** 1 Key Laboratory of Medicinal Chemistry and Molecular Diagnosis of Ministry of Education, Baoding 071002, China; 2 College of Life Science, Hebei University, Baoding 071002, China

**Keywords:** *Pestalotiopsis karstenii*, secondary metabolites, pestalrone A, pestalrone B

## Abstract

Two new oxysporone derivatives, pestalrone **A** (**1**) and pestalrone **B** (**2**), along with two known structurally related compounds **3**,**4**, were from the fermentation broth of the endophytic plant fungus *Pestalotiopsis karstenii* isolated from stems of *Camellia sasanqua*. Their structures and relative configurations were elucidated by extensive spectroscopic analysis and comparison of chemical shifts with related known compounds. Compound **2** exhibited significant activities agains HeLa, HepG2 and U-251 with IC_50_ values of 12.6, 31.7 and 5.4 µg/mL, respectively.

## 1. Introduction

**Figure 1:**
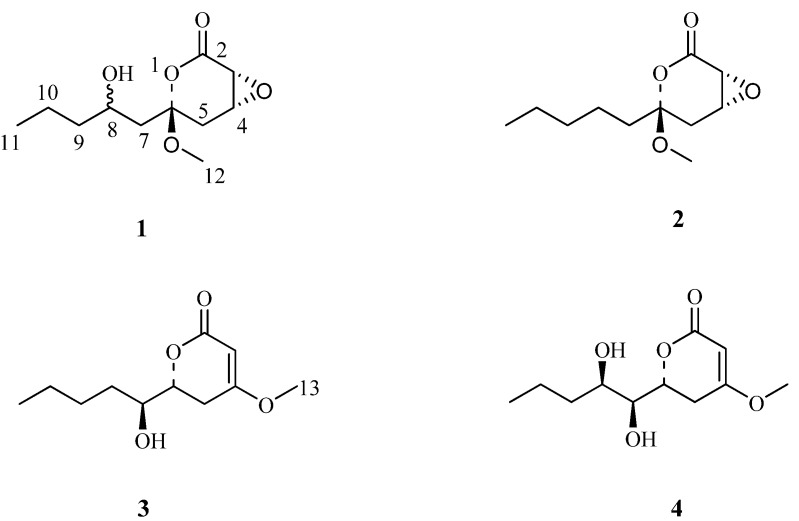
The structures of compounds **1**–**4**.

*Pestalotiopsis* species (family *Amphisphaeriaceae*) are widely distributed all over the World. Most of them are plant pathogens and many are saprobes in soil or in plant debris [1,2]. Previous investigations on some species of this genus have led to the isolation of numerous structurally diverse metabolites [3,4,5,6,7,8,9,10,11,12,13,14,15,16], including production of important compounds such as taxol [15,16]. During an ongoing search for new bioactive natural products from species of this genus, a strain of *Pestalotiopsis karstenii* isolated from stems of *Camellia sasanqua* collected from Nanning, Guangxi Province, China, was grown in a liquid fermentation culture which led to the isolation of four oxysporone derivatives, including two new ones. In this paper, we report the isolation and structural identification of the two new compounds, and the antitumor properties of compounds **1**–**4** (Figure 1).

## 2. Results and Discussion

**Figure 2:**
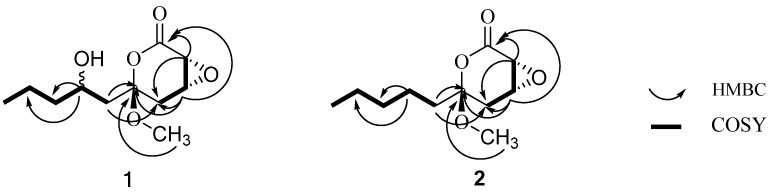
Selected HMBC and ^1^H-^1^H COSY correlations of pestalrone **A** (**1**) and pestalrone **B** (**2**).

The molecular formula of pestalrone A (**1**) was determined to be C_11_H_18_O_5_ from the accurate mass of the quasi-molecular ion peak at *m/z* 253.1038 ([M+Na]^+^) in its HR-ESI-MS spectrum, indicating three degrees of unsaturation. The IR spectrum revealed absorption bands of hydroxyl (3448 cm^−1^) and carbonyl (1735 cm^−1^) groups. The ^1^H-NMR spectrum (Table 1) indicated the presence of two methyl proton signals [δ*_H_* 0.96 (t, *J* = 7.2 Hz), 3.93 (s)], four methylene proton signals [δ*_H_* 1.88 (1H, dd, *J* = 13.7, 4.1 Hz), 2.42 (1H, m); 2.78 (1H, d, *J* = 18.6 Hz), 2.98 (1H, dd, *J* = 18.6, 2.7 Hz); 1.73 (1H, m), 1.39 (1H, m); 1.51 (2H, m)], and three oxygenated methine proton signal [δ*_H_* 3.65 (1H, dd, *J* = 2.8 Hz), 4.82 (1H, dd, *J* = 3.7, 1.7 Hz), 3.67 (1H, m)]. The ^13^C-NMR (DEPT) exhibited 11 carbon resonances, which were ascribable to two methyls (including one methoxy one), four methylenes, three oxygenated methines, one oxygenated quaternary carbon, and one carbonyl group. One carbonyl accounted for one of the three required degrees of unsaturation. The remaining degrees of unsaturation had to be present as two rings. Comparison of the NMR data of **1** with those of pestalotin (**3**), first isolated from *Pestalotia cryptomeriaecola* as a gibberellin synergist [17], revealed that **1** is composed of a tetrahydro-2*H*-pyran-2-one core attached with a long-chain subsituted alkyl group. The distinct differences between **1** and pestalotin (**3**) are: the chemical shift values of C-3, C-4, C-6, C-7 and C-8 of **1** [δC 76.4 (d, C-3), 71.7 (d, C-4), 95.7 (s, C-6), 40.7 (t, C-7), 66.2 (d, C-8)] are absent in pestalotin [δC 90.0 (d, C-3), 173.2 (s, C-4), 72.4 (d, C-6), 78.4 (d, C-7), 32.4 (t, C-8)]. In addition, the chemical shift value at C-2 (δC 169.2) in **1** was shifted downfield compared to pestalotin [δC 166.8 (s, C-2)] because of the double bond in pestalotin was reduced in **1**. Further interpretation of HMBC spectrum showed the following long-range correlations (Figure 2): H-3 with C-2 and C-5, H-4 with C-2 and C-6, H-5 with C-3. The above spectral evidences, along with the proton spin system: H-3/H-4/H-5 observed in ^1^H, ^1^H-COSY (Figure 2) correlations, led to the establishment of a tetrahydro-2H-pyran-2-one core unit. Similarly, a long-chain subsituted alkyl group was deduced from the correlations of H-7/H-8/H-9/H-10/H-11 observed in ^1^H, ^1^H-COSY, coupling with HMBC correlations from H-7 to C-9, from H-8 to C-10, and from H-11 to C-9. The linkage between a tetrahydro-2H-pyran-2-one core unit and a long-chain subsituted alkyl group was clearly detected from C-6 to C-7, since HMBC (Figure 2) correlations from H-7 to C-5 and from H-8 to C-6 were observed. Correlation of H-12 with C-6 located the methoxy group at C-6. Therefore, the planar structure of **1** was tentatively assigned as shown in Figure 1. 

**Table 1:** NMR spectroscopic data of pestalrone **A** (**1**) and pestalrone **B** (**2**) in chloroform-*d_6_*.

NO.	Pestalrone A (1)		Pestalrone B (2)	
	δ_H_^a^ ( *J* in Hz)	δ_C_^ b^	δ_H_^a^ ( *J* in Hz)	δ_C_^b^
2		169.2 (s)		169.0 (s)
3	3.65 (1H, d, *J* = 2.8)	76.4 (d)	4.17 (1H, d, 2.1)	83.1 (d)
4	4.82 (1H, dt, *J* = 4.0, 2.3)	71.7 (d)	4.80 (1H, t, *J* = 3.0)	78.8 (d)
5	1.88 (1H, dd, *J* = 13.7, 4.1) 2.42 (1H, m)	29.1 (t)	2.03 (1H, d, *J* = 12.5) 2.48 (1H, dt, 12.5, 2.9)	35.8 (t)
6		95.7 (s)		104.7 (s)
7	2.78 (1H, d, *J* = 18.6) 2.98 (1H, dd, *J* = 18.6, 2.7)	40.7 (t)	2.81 (1H, d, *J* = 17.9) 3.03 (1H, dd, *J* = 17.9, 2.1)	45.7 (t)
8	3.67 (1H, m)	66.2 (d)	1.61 (2H, m)	27.5 (t)
9	1.73 (1H, m) 1.39 (1H, m)	32.5 (t)	1.37 (2H, m)	30.5 (t)
10	1.51 (2H, m)	18.6 (t)	1.37 (2H, m)	22.6 (t)
11	0.96 (3H, t, *J* = 7.2)	14.0 (q)	0.92 (3H, m)	13.9 (q)
12	3.39 (3H, s)	48.9 (q)	3.43 (3H, s)	50.0 (q)

The relative configuration of **1** was determined by NOESY experiments, which showed significant correlations between H-4, H-3 and H_3_-12. These data suggested that these protons on the same face of the ring system. Due to small quantity of **1** available, we could not further determine the relative configuration at C(8) by chemical methods. Finally, on the basis of these data, the relative configuration of **1** was established as shown in Figure 1, and the compound was named pestalrone A (**1**).

The molecular formula of pestalrone B (2) was determined to be C_11_H_18_O_4_ by HR-ESI-MS (*m/z* 237.1088 [M+Na]^+^). Its ^1^H- and ^13^C-NMR spectrum showed resonances for two methyl groups (including one methoxy group), five methylenes, two oxygenated methines, one oxygenated sp3 quaternary carbon, and one carbonyl group. Careful analysis of NMR data, we found that the NMR spectral data of **2** was similar to those of **1** except for the absence of one hydroxyl group, that is, the hydroxyl group at C-8 of **1** [δC 66.2 (d)] is absent in **2** [δC 30.5 (t)]. This finding was evident from the ^1^H, ^1^H-COSY correlations between H-7/H-8 and HMBC correlations from H-8 to C-6 and C-10. On the basis of the above analysis, the structure of **2** was determined as shown in Figure 1.

The relative configuration of **2** was determined by NOESY experiments, which showed significant correlations between H-4, H-3 and H_3_-12. On the basis of these data, the relative configuration of **2** was established as shown in Figure 2, and the compound was named pestalrone B (**2**).

The two known compounds were identified as pestalotin (**3**) and hydroxypestalotin (**4**) by comparison of their spectroscopic data with literature data [17,18]. Compound **3** was first isolated from *Pestalotia cryptomeriaecola* as a gibberellin synergist and subsequently isolated from *Penicillium sp.* Compound **4** was previously isolated a culture filtrate of *Penicillium sp* which exhibited antiprotozoal activity.

Compounds **1**–**4** were evaluated for cytotoxic activity against five human tumor cell lines (HeLa, U-251, A549, HepG2 and MCF-7) with DDP as positive control. Compound **1** has no inhibitory effects on the five human tumor cell lines. Compound **2** exhibited significant activities against HeLa, HepG2 and U-251, with IC_50_ values of 12.6, 31.7 and 5.4 µg/mL, respectively, whereas **2** did not show noticeable cytotoxic activities against A549 and MCF-7. Compounds **3** and **4** displayed strong activies against U-251, with IC_50_ values of 2.5 and 12.0 µg/mL, respectively and no activities against HeLa, A549, HepG2 and MCF-7.

## 3. Experimental

### 3.1. General

Optical rotations: Perkin-Elmer 341 spectropolarimeter. IR spectra: Perkin-Elmer 577 spectrometer; KBr pellets; in cm^−1^. UV spectra: UV-210 spectrometer, λ_max_ (log ε) in nm. NMR spectra: Bruker AM-600 spectrometer; δ in ppm, J in Hz; Me4Si as internal standard. FT-MS spectra: Bruker apex-ultra 7.0 T spectrometer in *m/z*. Column chromatography (CC): silica gel (200~300 mesh, Yantai Zhi Fu Chemical Co., Ltd., China), TLC: silica gel GF254 plates (Yantai Zhi Fu Chemical Co., Ltd, China) and Sephadex LH-20 gel (25~100 μm, GE Healthcare Co., Ltd., Sweden).

### 3.2. Fungal Material and Cultivation Conditions

*Pestalotiopsis karstenii* was isolated from the stems of *Camellia sasanqua* in Nanning, Guangxi Province, China, in 2003, identified by Prof. Jing-Ze Zhang of Zhejiang University, and assigned the accession number L010 in the culture collection at College of Life Science, Key Laboratory of Medicinal Chemistry and Molecular Diagnosis of Ministry of Education, Hebei University. The fungal strain was cultured on slants of potato dextrose agar (PDA) at 28 °C for 10 days, and then inoculated into 500 mL Erlenmeyer flasks, each containing 200 mL of media (40 g of glucose, 400 g of potato (peeled), 6 g of KH_2_PO_4_, 3 g of MgSO_4_, 0.2 g of citric acid and 0.04 g of thiamin hydrochloride in 200 mL deionized H_2_O). The final pH of the media was adjusted to 6.5 before sterilization. Eighty 500 mL Erlenmeyer flasks, each containing 200 mL of liquid media were individually inoculated with 20 mL of the seed culture on a rotary shaker for 30 days. 

### 3.3. Extraction and Isolation

The fermented material was extracted with ethyl acetate for three times. The organic solvent was evaporated to dryness under vacuum to afford a crude extract (10 g), which was chromatographed by silica gel column chromatography (CC) using petroleum ether/ethyl acetate [100:0, 98:2, 95:5, 90:10, 80:20, 50:50 (v/v)] to afford six fractions (*frs.*) *1–6*. *Fr.3* (1.0 g) eluted with petroleum ether/ethyl acetate (95:5) was further purified by CC (silica gel; petroleum ether/ethyl acetate, 10:1) to afford five fractions (*frs.3.1*–*3.5*). *Fr.3.2* (200 mg) was separated by Sephadex LH-20 chromatograpy using MeOH as eluent to afford the compound **2** (6 mg). *Fr.5* (1.5 g) eluted with petroleum ether/ethyl acetate (80:20) was further purified by CC (silica gel; petroleum ether/ethyl acetate, 10:1) to afford seven fractions (*frs.5.1–5.7*). *Fr. 5.2* was separated by Sephadex LH-20 chromatograpy using chloroform/ methanol (1:1) as eluent and then preparative TLC (chloroform/acetone/methanol, 15:1:1) was used to afford the compound **3** (10 mg). *Fr.5.4* was future purified by Sephadex LH-20 chromatograpy using MeOH as eluent and then uses preparative TLC (chloroform/acetone, 5:1) to afford compound **1** (4 mg). *Fr.6* (0.8 g) was further purified by CC (silica gel; petroleum ether/ethyl acetate, 10:3) to afford three fractions *6.1–6.3*. *Fr.6.1* gave a crystalline residue which on recrystallization from ethyl acetate gave compound **4** (9 mg).

*Pestalrone* *A* (**1**): Isolated as pale yellow oil, [α]_D_^19^ = +24° (c = 4.4, CHCl_3_). IR (KBr) v_max_: 3448 (OH), 2958, 1735 (C=O), 1062 cm^−1^. UV (CHCl_3_) λ_max_ (lg ε): 210 (5.00) nm. ^1^H- and ^13^C-NMR, see Table 1. Positive ion HR-ESI-MS [M+Na]^+^ *m/z* 253.1038 (calcd for C_11_H_18_NaO_5_, 253.1038).

*Pestalrone* *B* (**2**): Isolated as pale yellow oil, [α]_D_^19^ = +56°, (c = 4.7, CHCl_3_). IR (KBr) v_max_: 2850, 2360, 1746 (C=O) cm^−1^. UV (CHCl_3_) λ_max_ (lg ε): 231 (3.72) nm, ^1^H- and ^13^C-NMR, see Table 1. Positive ion HR-APCI-MS [M+Na]^+^ *m/z* 237.1088 (calcd for C_11_H_18_NaO_4_, 237.1088).

### 3.4. Biological Assays

HeLa, U-251, A549, HepG2 and MCF-7 cell lines were grown in RPMI-1640 medium (GIBCO) supplemented with 10% heat-inactivated bovine serum, 2 nM L-glutamine, 105 IU/L penicillin, 100 mg/L streptomycin and 10 mM HEPES, pH 7.4. Cell was kept at 37 °C in a humidified 5% CO_2_ incubator. Growth inhibition of compounds **1**–**4**, with DDP as positive controls on HeLa, U-251, A549, HepG2 and MCF-7 cells were measured by the microculture tetrazolium (MTT) assay with minor modifications [19,20,21].

## 4. Conclusions

On the whole, we have isolated two new oxysporone derivatives, named pestalrone A (**1**) and pestalrone B (**2**), together with two known compounds **3**,**4** from the fermentation broth of the endophytic plant fungus *Pestalotiopsis karstenii*. Compounds **2**,**3** and **4** showed cytotoxic activity against HeLa (human cervical cancer cell line) and U-251 (human glioma cell line). 

## Supplementary Materials

Supplementary materials can be assessed at: http://www.mdpi.com/1420-3049/17/7/8554/s1.
