# Supplementary material for: Two New Oxysporone Derivatives from the Fermentation Broth of the Endophytic Plant Fungus Pestalotiopsis karstenii Isolated from Stems of Camellia sasanqua
Source: Molecules. 2012 Jul 17;17(7):8554–60. doi: 10.3390/molecules17078554 (PMC6268523; doi:10.3390/molecules17078554)

The HR-ESI-MS Spectrum of Compound **1**.

Mass Spectrum SmartFormula Report

Analysis Info

Analysis Name	D:\Data\User\20111019\ZL_7_ESI_POS_000002.d	Acquisition Date	10/19/2011 4:43:20 PM
Method	Metal_Trypsin digestion	Operator	
Sample Name	ZL_7_ESI_POS	Instrument	apex-Ultra
Comment	ZL_7_ESI_POS		

Acquisition Parameter

Polarity	Positive	Source	ESI	No. of Laser Shots	20
Averaged Scans	4	No. of Cell Fills	1	Laser Power	51.0 %
Broadband Low Mass	100.3 m/z	End Plate	3500.0 V	MALDI Plate	300.0 V
Broadband High Mass	2000.0 m/z	Capillary Entrance	4000.0 V	Imaging Spot Diameter	2000.0 μm
Acquisition Mode	Single MS	Skimmer 1	20.0 V		
Pulse Program	basic	Drying Gas Temperature	180.0 °C	Calibration Date	Wed Oct 19 09:26:51
Source Accumulation	0.0 sec	Drying Gas Flow Rate	4.0 L/min	Data Acquisition Size	201072
Ion Accumulation Time	0.0 sec	Nebulizer Gas Flow Rate	1.0 L/min	Apodization	Sine-Bell Multiplication
Flight Time to Acq. Cell	0.0 sec				

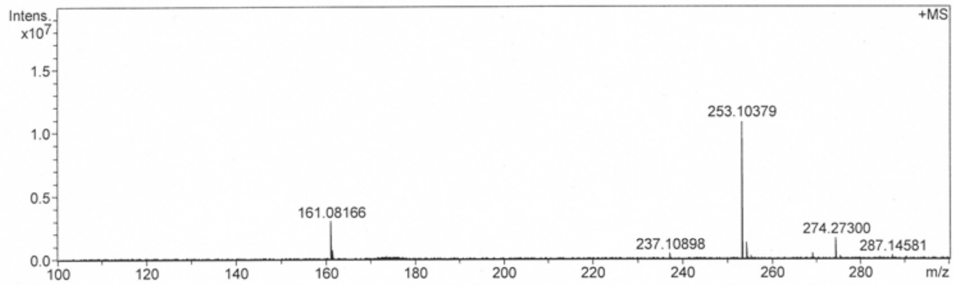

Meas. m/z	#	Formula	Score	m/z	err [mDa]	err [ppm]	mSigma	rdb	e⁻ Conf	N-Rule
253.10379	1	C 11 H 18 Na O 5	100.00	253.10464	0.9	3.4	6.3	2.5	even	ok

The ^{1}H-NMR Spectrum of Compound **1**.

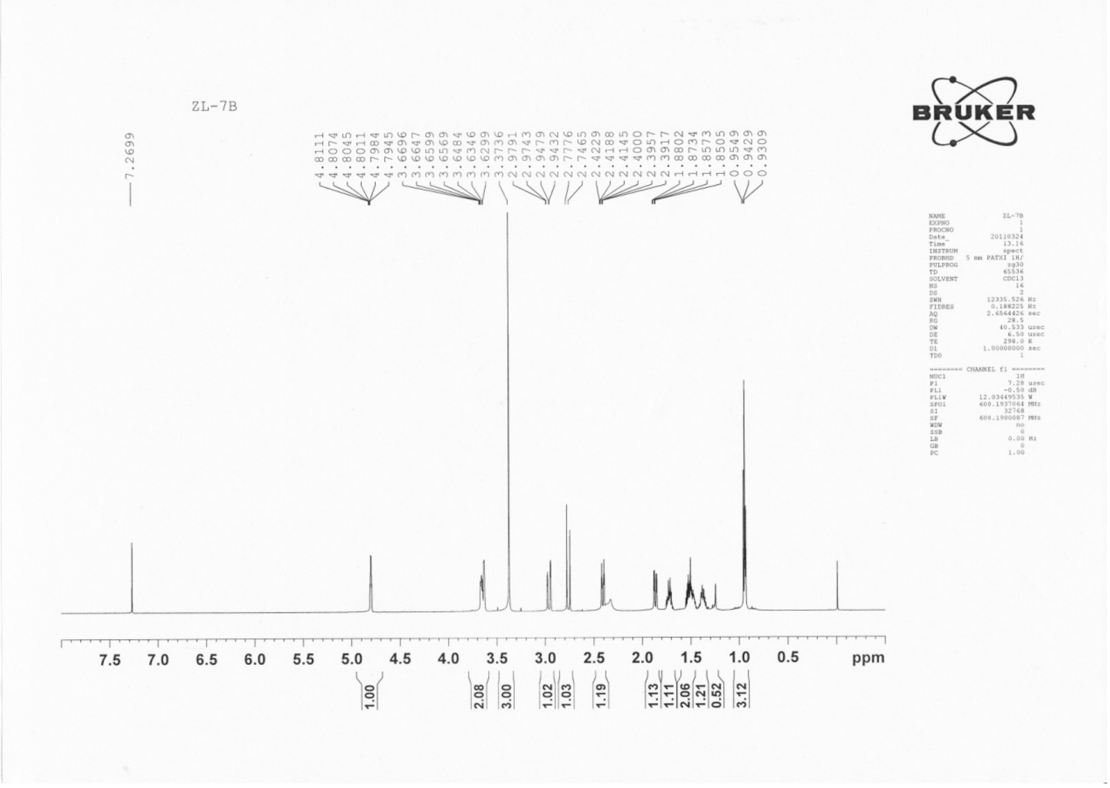

The ^{13}C-NMR Spectrum of Compound **1**.

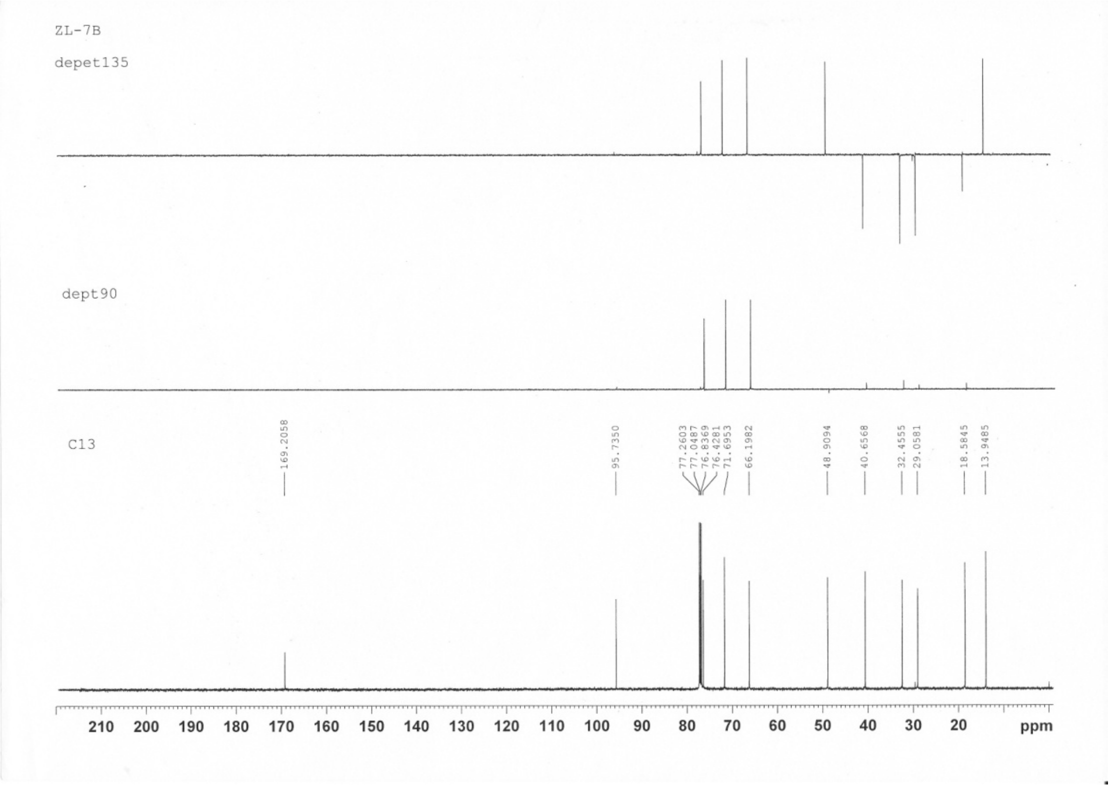

The HSQC Spectrum of Compound **1**.

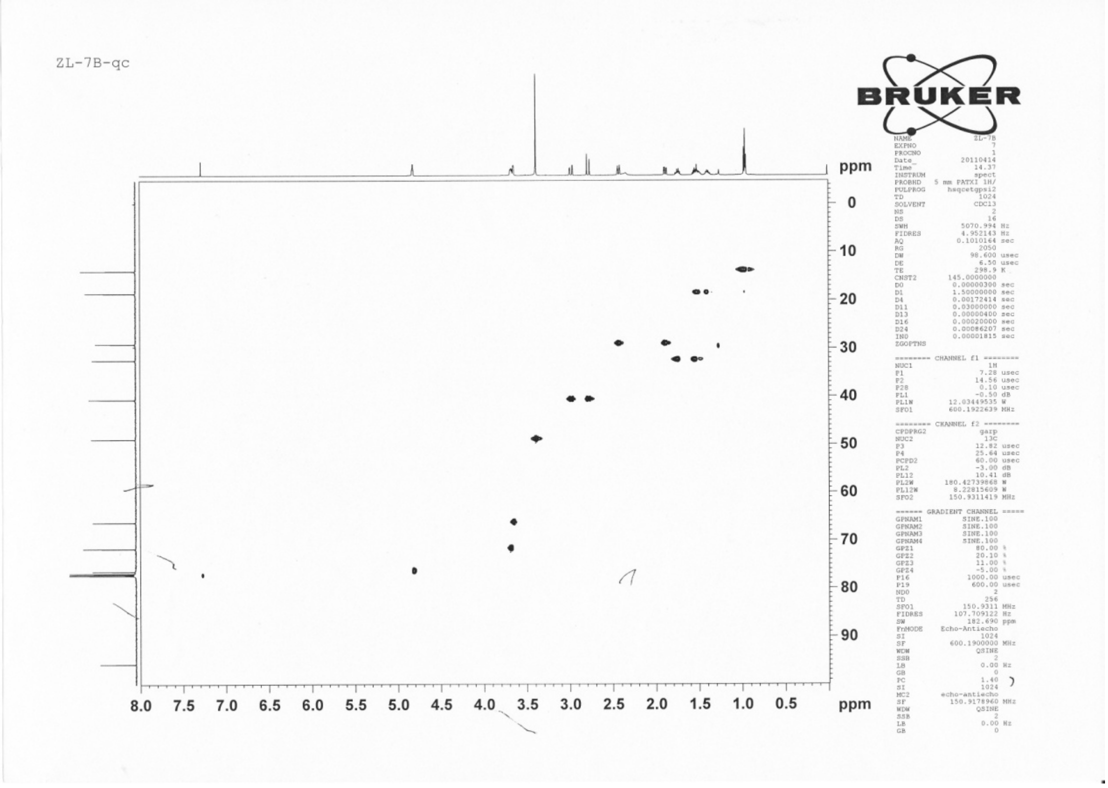

The ^{1}H-^{1}H COSY spectrum of compound **1**.

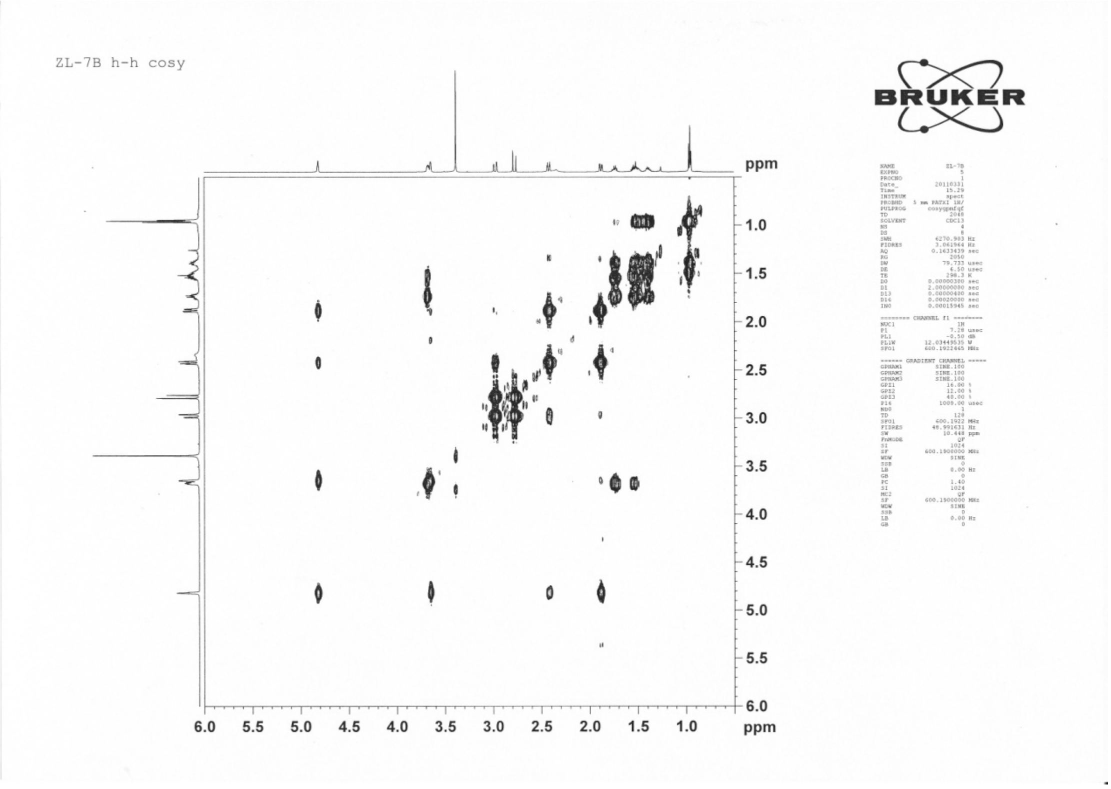

The HMBC Spectrum of Compound **1**.

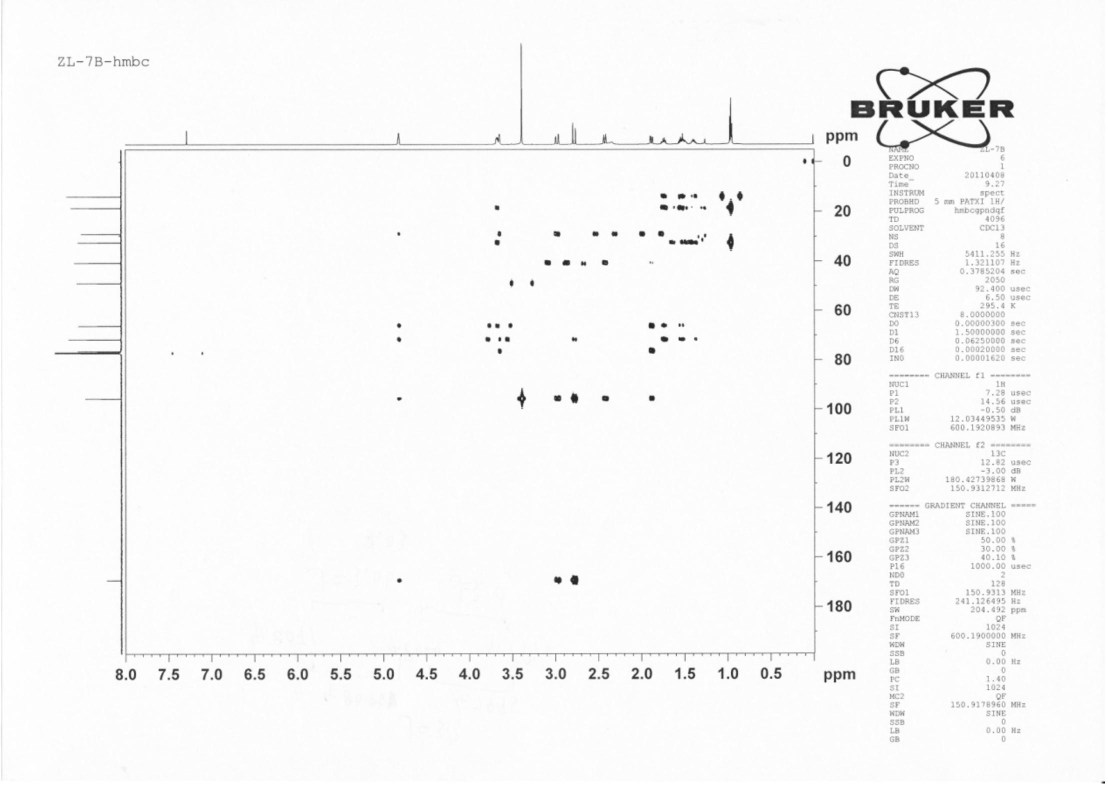

The NOESY Spectrum of Compound **1**.

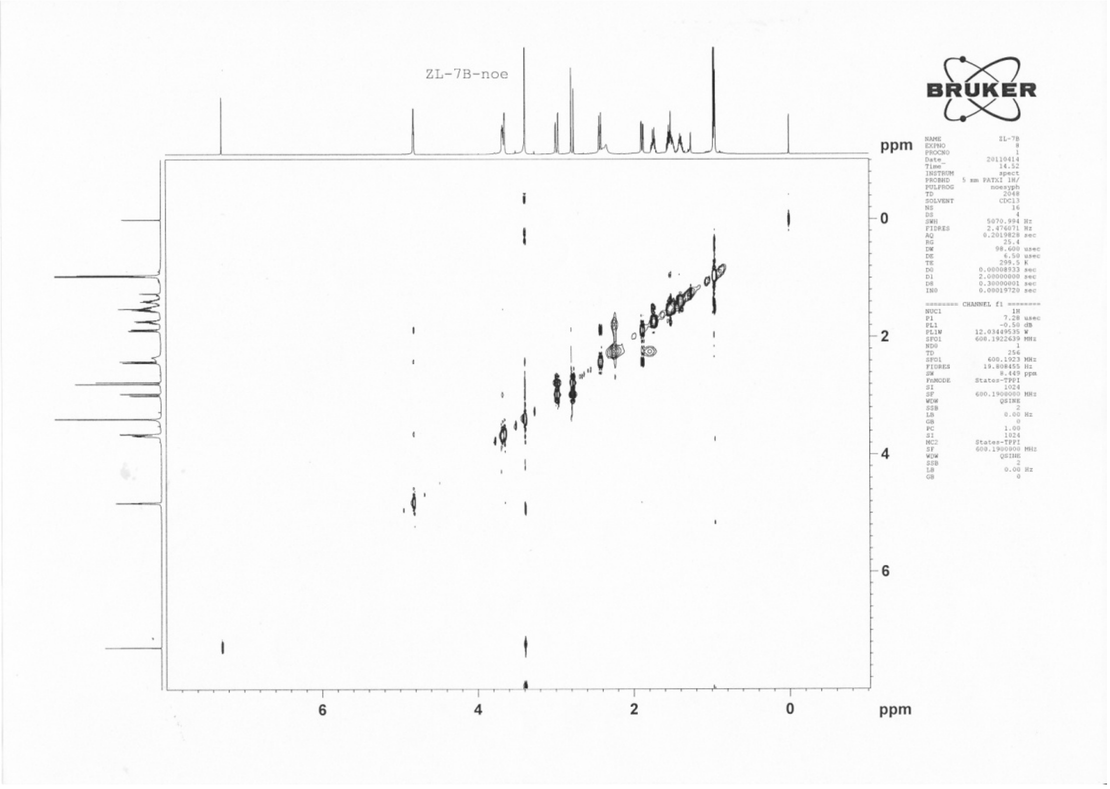

The IR Spectrum of Compound **1**.

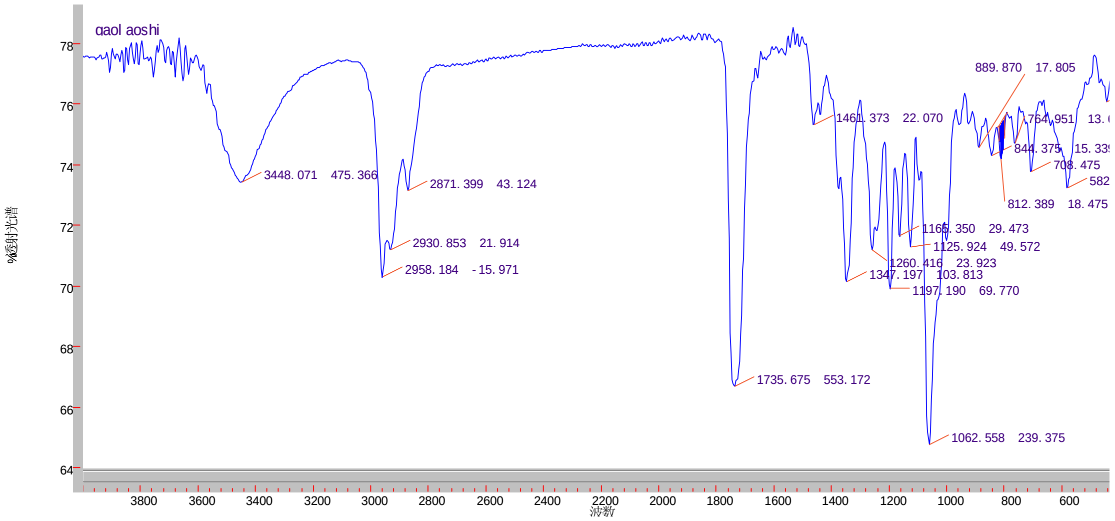

The HR-ESI-MS Spectrum of Compound **2**.

Mass Spectrum SmartFormula Report

Analysis Info

Analysis Name	D:\Data\User\20111019\ZL_10_ESI_POS_000003.d	Acquisition Date	10/19/2011 4:41:52 PM
Method	Metal_Trypsin digestion	Operator	
Sample Name	ZL_10_ESI_POS	Instrument	apex-Ultra
Comment	ZL_10_ESI_POS		

Acquisition Parameter

Polarity	Positive	Source	ESI	No. of Laser Shots	20
Averaged Scans	4	No. of Cell Fills	1	Laser Power	51.0 %
Broadband Low Mass	100.3 m/z	End Plate	3500.0 V	MALDI Plate	300.0 V
Broadband High Mass	2000.0 m/z	Capillary Entrance	4000.0 V	Imaging Spot Diameter	2000.0 μm
Acquisition Mode	Single MS	Skimmer 1	20.0 V		
Pulse Program	basic	Drying Gas Temperature	180.0 °C	Calibration Date	Wed Oct 19 09:26:51
Source Accumulation	0.0 sec	Drying Gas Flow Rate	4.0 L/min	Data Acquisition Size	201072
Ion Accumulation Time	0.0 sec	Nebulizer Gas Flow Rate	1.0 L/min	Apodization	Sine-Bell Multiplication
Flight Time to Acq. Cell	0.0 sec				

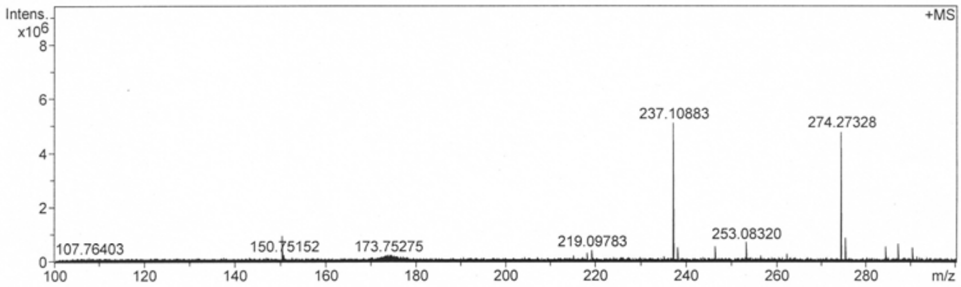

Meas. m/z	#	Formula	Score	m/z	err [mDa]	err [ppm]	mSigma	rdb	e¯ Conf	N-Rule
237.10883	1	C 11 H 18 Na O 4	100.00	237.10973	0.9	3.8	11.4	2.5	even	ok

Bruker Compass DataAnalysis 4.0 printed: 10/26/2011 10:20:05 AM Page 1 of 1

The ^{1}H-NMR Spectrum of Compound **2**.

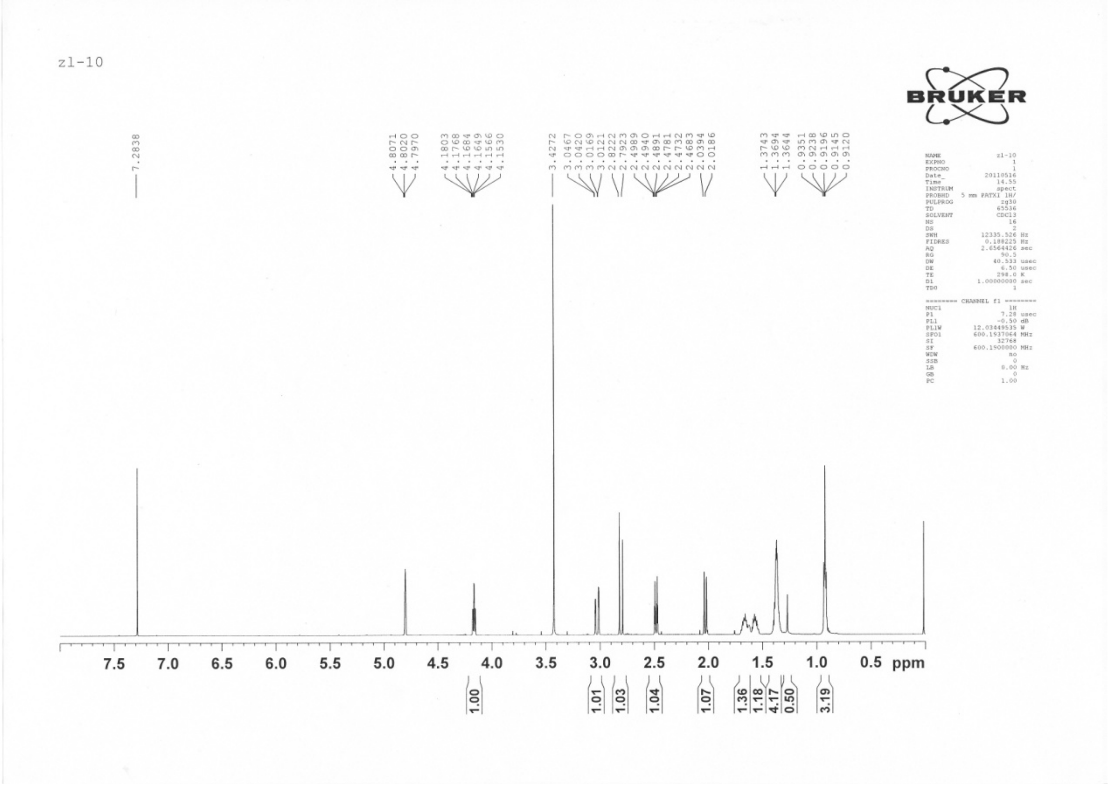

The ^{13}C-NMR of compound **2**.

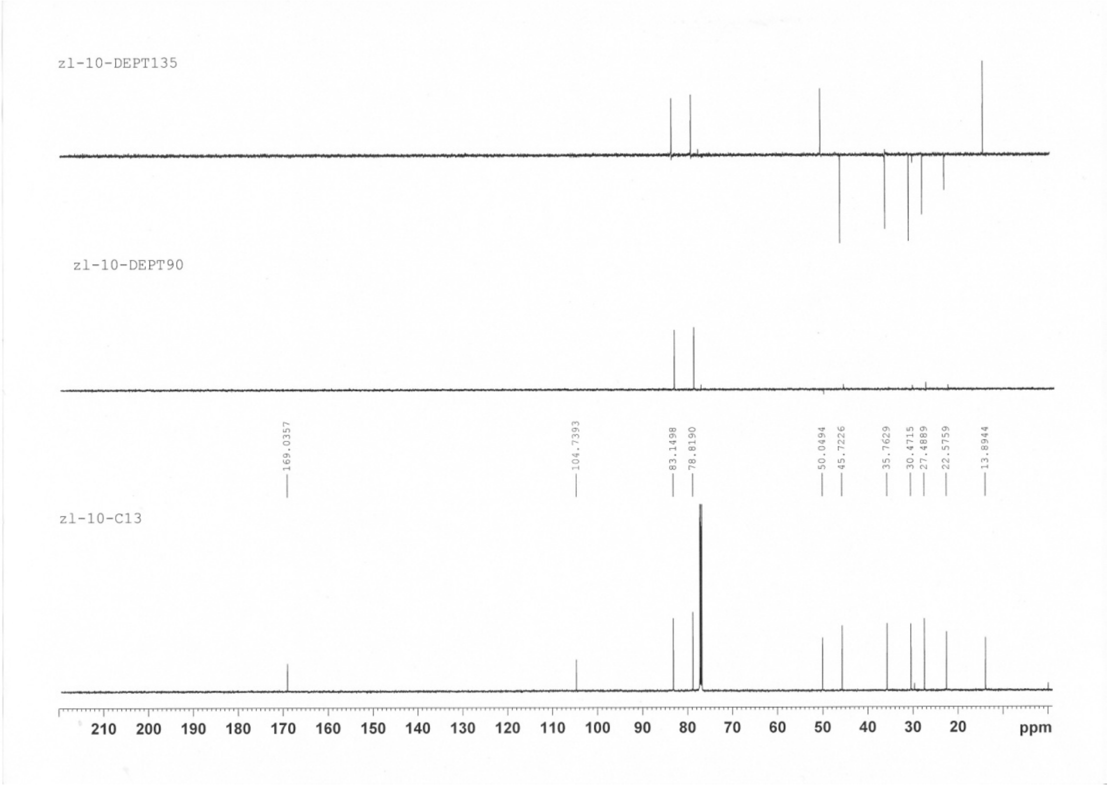

The HSQC Spectrum of Compound **2**.

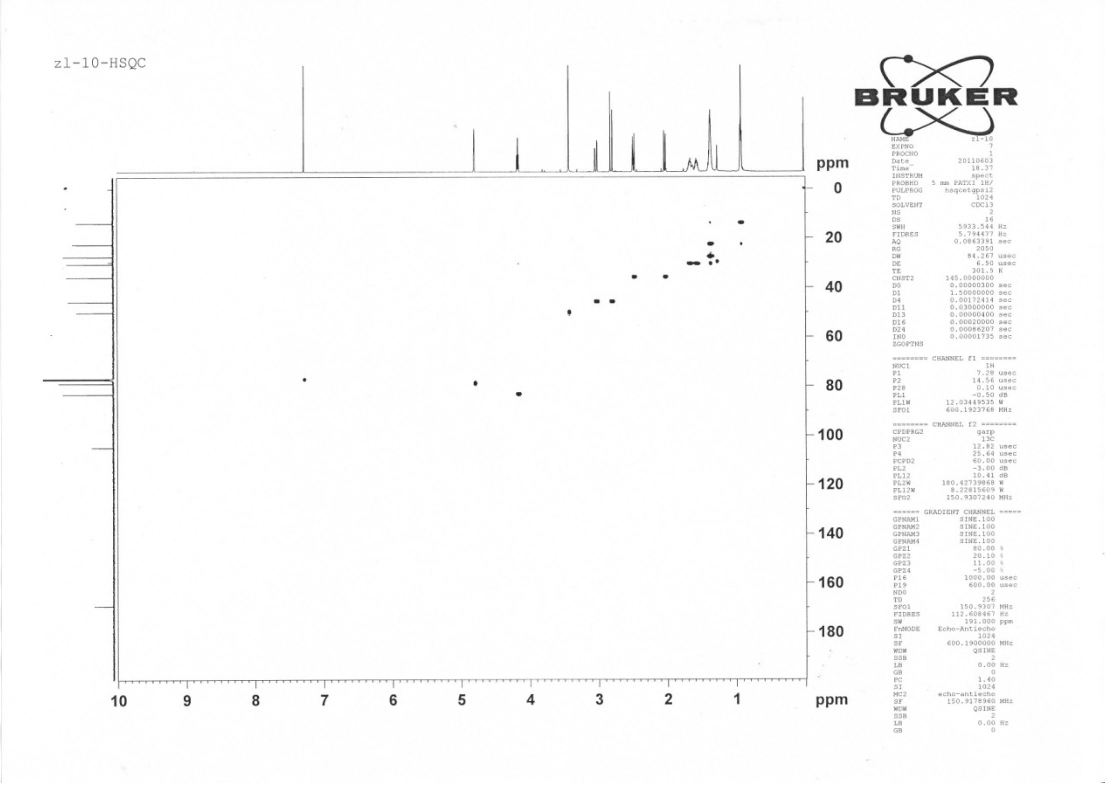

The ^1H-^1H COSY Spectrum of Compound **2**.

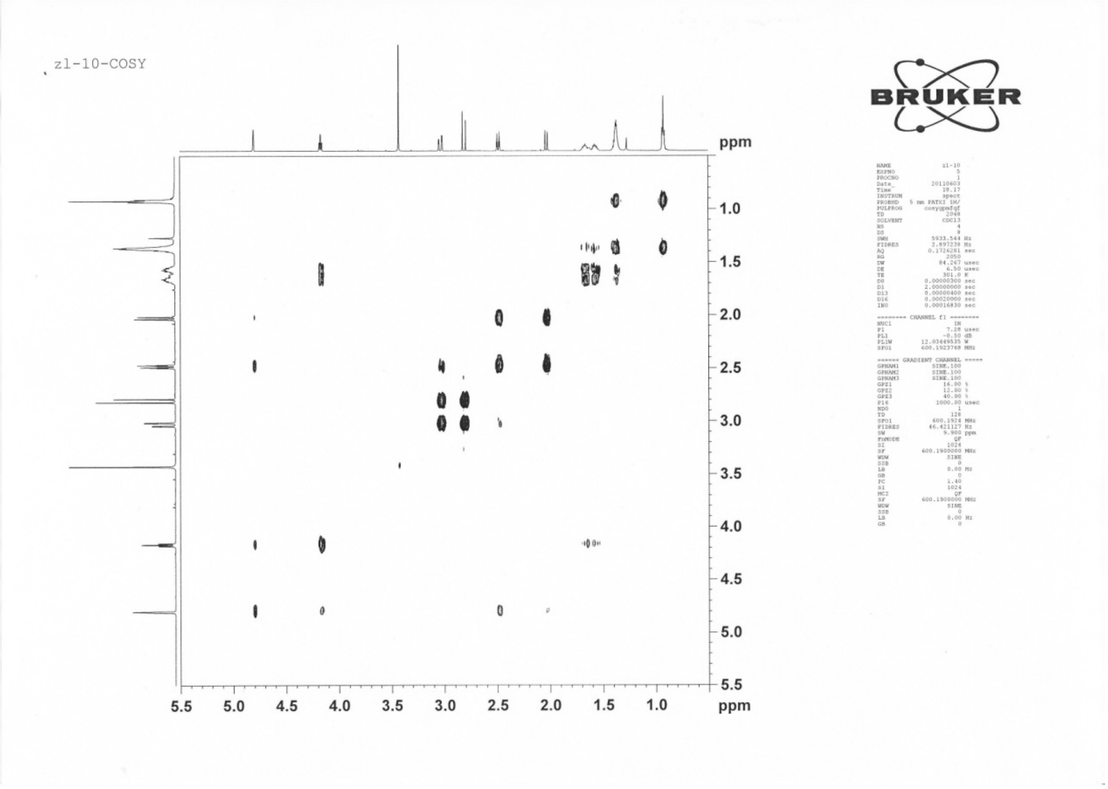

The HMBC Spectrum of Compound **2**.

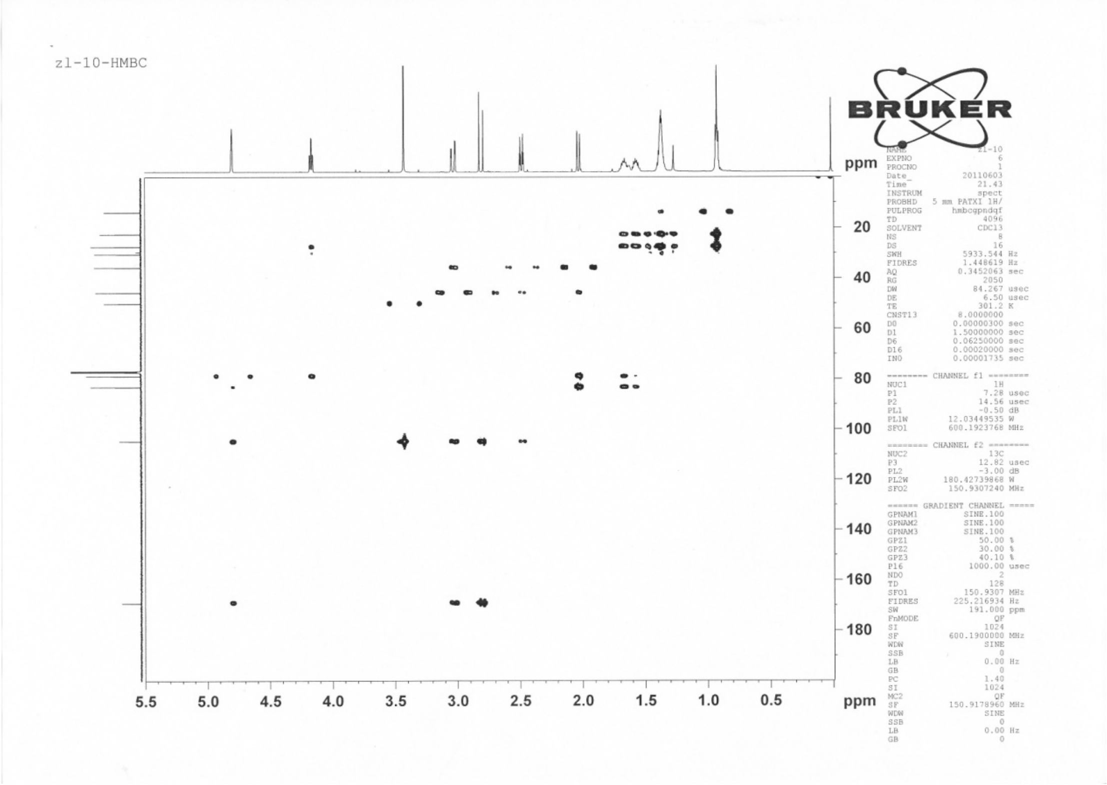

The NOESY Spectrum of Compound **2**.

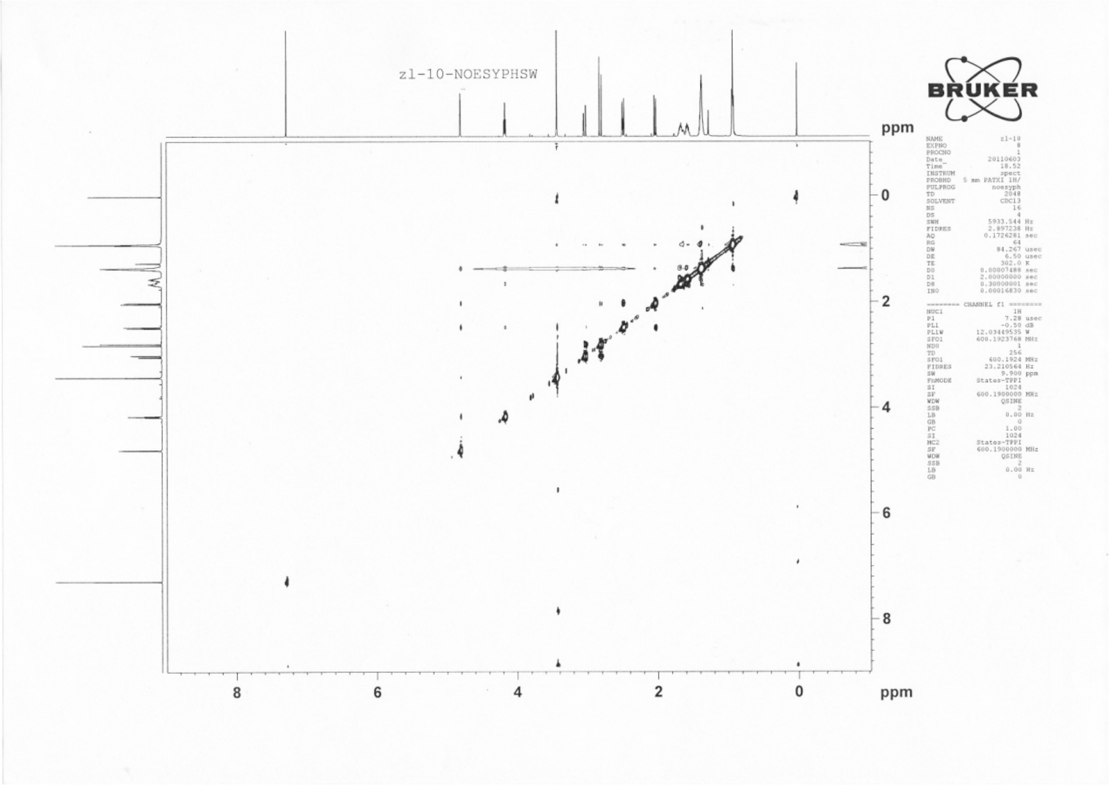

The IR Spectrum of Compound **2**.

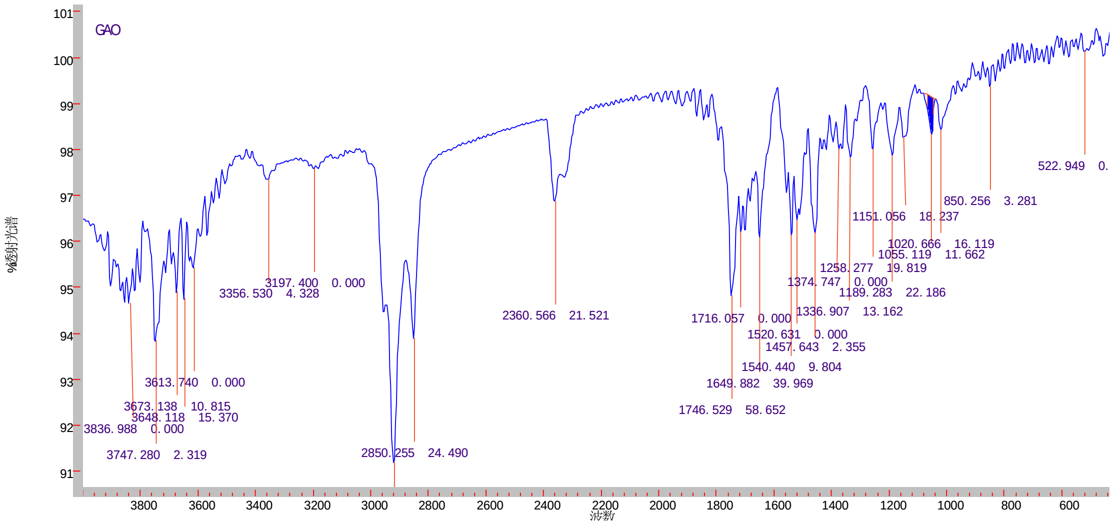

Supplement: Supplementary file 1 [file molecules-17-08554-s001.pdf]
